# Evaluating the Performance of DeepSeek-R1 and DeepSeek-V3 Versus OpenAI Models in the Chinese National Medical Licensing Examination: Cross-Sectional Comparative Study

**DOI:** 10.2196/73469

**Published:** 2025-11-14

**Authors:** Weiping Wang, Yuchen Zhou, Jingxuan Fu, Ke Hu

**Affiliations:** 1 Department of Radiation Oncology Peking Union Medical College Hospital Chinese Academy of Medical Sciences & Peking Union Medical College Beijing China; 2 Tsinghua Medicine School of Medicine Tsinghua University Beijing China; 3 Department of clinical laboratory Xuanwu Hospital Capital Medical University Beijing China

**Keywords:** large language models, DeepSeek, OpenAI, Chinese Medical Licensing Examination, artificial intelligence, AI

## Abstract

**Background:**

Deepseek-R1, an open-source large language model (LLM), has generated significant global interest in the past months.

**Objective:**

This study aimed to compare the performance of DeepSeek and OpenAI LLMs on the Chinese National Medical Licensing Examination (NMLE) and evaluate their potential in medical education.

**Methods:**

This cross-sectional study assessed 2 DeepSeek models (DeepSeek-R1 and DeepSeek-V3), 3 OpenAI models (ChatGPT-o1 pro, ChatGPT-o3 mini, and GPT-4o), and 2 additional Chinese LLMs (ERNIE 4.5 Turbo and Qwen 3) using the 2021 NMLE. Model performance was evaluated based on overall accuracy, accuracy across question types (A1, A2, A3 and A4, and B1), case analysis and non–case analysis questions, medical specialties, and accuracy consensus between different model combinations.

**Results:**

All LLMs successfully passed the NMLE. DeepSeek-R1 achieved the highest accuracy (573/597, 96%), followed by DeepSeek-V3 (558/600, 93%), both of which significantly outperformed ChatGPT-o1 pro (450/600, 75%), ChatGPT-o3 mini (455/600, 75.8%), and GPT-4o (452/600, 75.3%; *P*<.001 for all comparisons). Performance disparities were consistent across various question types (A1, A2, A3 and A4, and B1), case analysis and non–case analysis questions, different types of case analyses, and medical specialties. The accuracy consensus between DeepSeek-R1 and DeepSeek-V3 reached 97.7% (544/557), significantly outperforming DeepSeek-R1 alone (*P*=.04). Two additional Chinese LLMs, ERNIE 4.5 Turbo (572/600, 95.3%) and Qwen 3 (555/600, 92.5%), also exhibited significantly better performance compared to the 3 OpenAI models (all *P*<.001).

**Conclusions:**

This study demonstrates that DeepSeek-R1 and DeepSeek-V3 significantly outperform OpenAI models on the NMLE. DeepSeek models show promise as tools for medical education and exam preparation in the Chinese language.

## Introduction

DeepSeek-R1, an open-source large language model (LLM), has generated significant global interest in the past months [1-5]. Launched by DeepSeek on January 20, 2025, this model demonstrates a performance comparable to that of OpenAI’s ChatGPT-o1 in tasks involving mathematics, coding, and reasoning [2]. By leveraging a “mixture of experts” architecture, DeepSeek reduces the computational resources required for model training while enhancing the efficiency of query responses [6]. In addition, its lower cost and interface fees make it a more accessible option for users, providing a cost-effective alternative to OpenAI models. As an open-source LLM, DeepSeek-R1 offers users the ability to view and modify its source code, encouraging further improvements and customization. The release of this model has already made a splash in academic circles [1-3].

In recent years, OpenAI has maintained a leading position in the field of LLMs. OpenAI recently launched the ChatGPT-o1 pro and ChatGPT-o3 mini models, which are widely regarded as among the most powerful models available. GPT-4o, launched in May 2024, represents OpenAI’s best-performing free model, with extensive applications and robust validation over the past year [7,8].

LLMs hold substantial potential in the medical domain, including clinical decision support [9,10], medical image analysis [11,12], health education, patient counseling, and medical training [13]. Medical licensing examinations are a critical entry test for medical licensure, and successfully passing them indicates a foundational understanding of medical knowledge. A model’s ability to pass these exams not only suggests its potential to aid medical students in their exam preparation but also reflects its proficiency in medical knowledge and reasoning—skills essential for clinical decision-making and medical image interpretation.

Studies have shown that the GPT-4o model achieves an accuracy of 90.4% on the US Medical Licensing Examination (USMLE) [7], whereas its performance on the Chinese National Medical Licensing Examination (NMLE) stands at 84.9%, notably lower than its performance on the USMLE, as well as that on the United Kingdom’s Professional and Linguistic Assessments Board (PLAB) test (93.3%) and the Hong Kong Medical Licensing Examination (91.7%) [8]. Developed by a Chinese company, the DeepSeek-R1 and DeepSeek-V3 models feature a higher proportion of Chinese-language corpora, making their performance on Chinese-language exams particularly promising.

In this study, we compared the performance of the DeepSeek and OpenAI LLMs on the NMLE and evaluated their potential in Chinese medical education.

## Methods

### LLMs Evaluated in This Study

The models evaluated in this study were DeepSeek-R1, DeepSeek-V3, ChatGPT-o1 pro, ChatGPT-o3 mini, GPT-4o, ERNIE 4.5 Turbo, and Qwen 3. DeepSeek-R1 and DeepSeek-V3 were developed by DeepSeek AI. ChatGPT-o1 pro, ChatGPT-o3 mini, and GPT-4o were developed by OpenAI. ERNIE 4.5 Turbo was developed by Baidu, and Qwen 3 was developed by the Alibaba Group.

### NMLE Description

The NMLE is a comprehensive, nationwide exam required for obtaining medical practice credentials in China. All questions in the exam are multiple choice in Chinese, with 5 options provided and only 1 option being correct. The exam is divided into 4 sections, each consisting of 150 multiple-choice questions. The total number of questions is 600, with each being worth 1 point. To pass the exam, candidates must achieve a score of ≥360 points. Each section is allotted 2.5 hours, and the entire exam lasts 10 hours in total.

The questions in the exam are classified into 4 types. A1-type questions assess basic medical knowledge, and A2-, A3-, and A4-type questions are case analysis questions. The A2-type questions are presented with a single case, followed by 1 question related to that case. A3 and A4–type questions also follow a case-based format: each case is accompanied by 2 to 4 associated questions. B1-type questions consist of several sets of questions, each with a common list of 5 options. Some options may be selected once, multiple times, or not at all, depending on the question.

The 2021 NMLE was used in this study [14]. The Chinese National Medical Examination Center, which administered this NMLE, has confirmed that the use of these questions for academic research is exempt from copyright restrictions.

### Model Testing Procedure

All models were assessed using their publicly accessible web interfaces in default configurations, precluding manual adjustment of parameters such as temperature, maximum tokens, or system prompts. This study used standardized prompting without in-context learning. All models were evaluated using identical input prompts for each question type (provided in Multimedia Appendix 1), with no additional examples or contextual demonstrations provided during testing. A standard prompt in Chinese was copied into the conversation window of each model to set the conditions for the exam. For the A1- and A2-type questions, 1 question was copied into the chat window at a time, and the models responded individually to each question. For A3 and A4–type questions, all related questions for a single case were input into the chat window simultaneously, and the models provided answers to all questions associated with that case. For the B1-type questions, all questions in a set with a shared list of 5 possible answers were input at once, and the models answered all questions in the set. Each set of questions (or individual question for the A1 and A2 types) was processed in a new, blank chat window. Testing took place from February 3, 2025, to February 9, 2025.

In addition, 2 experienced attending physicians independently classified the case-based questions (A2, A3, and A4 types) using 2 different methods. The first method involved categorizing the questions into broad categories such as examination, diagnosis, treatment, and other relevant areas. The second method involved classifying the questions according to medical specialty, including internal medicine, surgery, obstetrics and gynecology, pediatrics, neurology, psychiatry, emergency medicine, and others. For cases involving multiple specialties, the primary specialty was determined based on the most relevant medical department. In instances of disagreement between the 2 attending physicians, a senior physician made the final decision regarding the classification.

To investigate the underlying factors contributing to DeepSeek models’ superior performance, we conducted parallel evaluations by translating both the NMLE question bank and standardized prompts into English (see Multimedia Appendix 1 for the English-language prompt versions). GPT-4o was systematically evaluated using both the original Chinese questions and their English-translated counterparts to enable direct performance comparison across language conditions.

### Statistics

Questions that the models did not answer were marked as omitted and excluded from the accuracy calculation. Accuracy consensus was defined as the ratio of agreement on correct responses between 2 models relative to their overall agreement (ie, considering both correct and incorrect responses).

For paired-sample comparisons, we used the McNemar chi-square test for 2-group comparisons and the Cochran *Q* test for multiple-group comparisons. When analyzing independent samples, we used the Pearson chi-square test, continuity correction, or the Fisher exact test as appropriate. The κ consistency test was used to quantitatively assess the concordance between 2 models. In terms of the κ coefficients, values exceeding 0.75 are considered to indicate excellent agreement, values within the range of 0.40 to 0.75 suggest good agreement, whereas values below 0.40 are indicative of poor agreement.

All statistical analyses were conducted using SPSS (version 23.0; IBM Corp), and a 2-tailed *P* value of <.05 was considered statistically significant.

## Results

### Overall Accuracy

DeepSeek-R1 did not provide answers for 3 questions, responding with “Sorry, I haven’t yet learned how to approach these types of questions. I specialize in math, coding, and logic-related problems. Feel free to engage with me on these topics” (Multimedia Appendix 1). These 3 questions were marked as omissions. The remaining 4 models answered all the questions.

**Figure 1:**
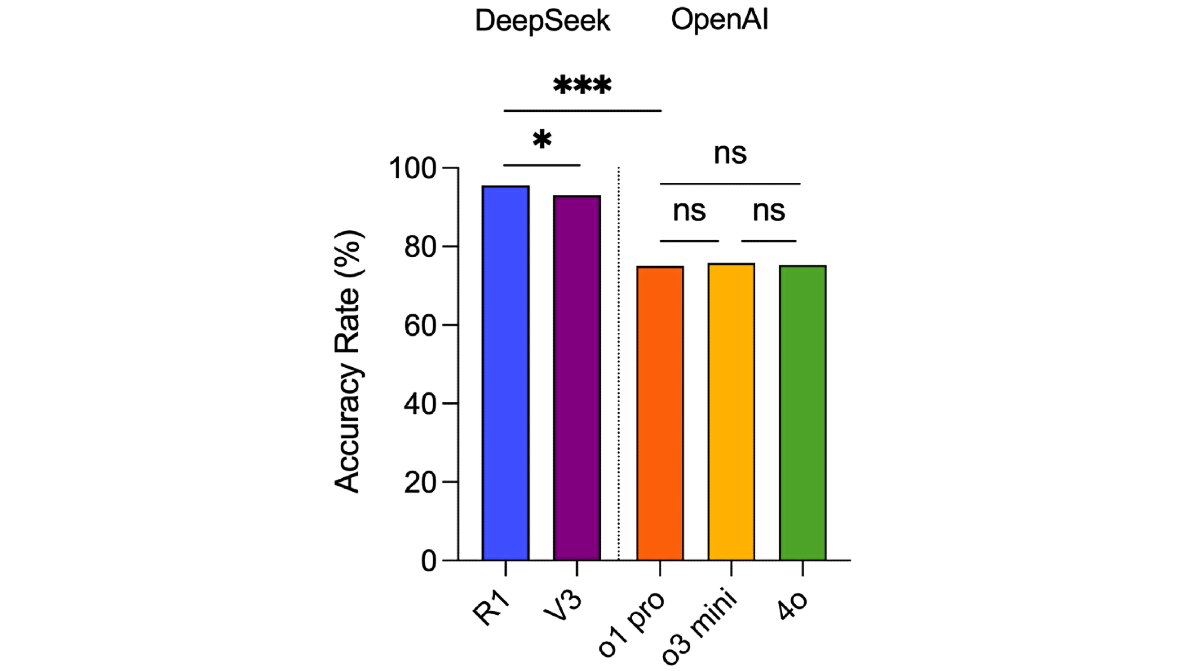
Overall accuracy rates of DeepSeek-R1, DeepSeek-V3, ChatGPT-o1 pro, ChatGPT-o3 mini, and GPT-4o. * *P*<.05, *** *P*<.005, and ns (no significance).

DeepSeek-R1, DeepSeek-V3, ChatGPT-o1 pro, ChatGPT-o3 mini, and GPT-4o answered 573, 558, 450, 455, and 452 questions correctly, respectively, achieving accuracy rates of 96% (573/597), 93% (558/600), 75% (450/600), 75.8% (455/600), and 75.3% (452/600), respectively (Figure 1 and Multimedia Appendix 1). The 3 questions that DeepSeek-R1 did not answer were excluded from the calculation.

All models scored above 360 points, thereby passing the NMLE. The accuracy of DeepSeek-R1 and DeepSeek-V3 was significantly higher than that of ChatGPT-o1, ChatGPT-o3, and GPT-4o (*P*<.001 for all comparisons). Between models from the same developers, DeepSeek-R1’s accuracy was significantly higher than that of DeepSeek-V3 (*P*=.02), whereas no substantial differences were observed between ChatGPT-o1 pro, ChatGPT-o3 mini, and GPT-4o.

### Accuracy Across Different Question Types

**Figure 2:**
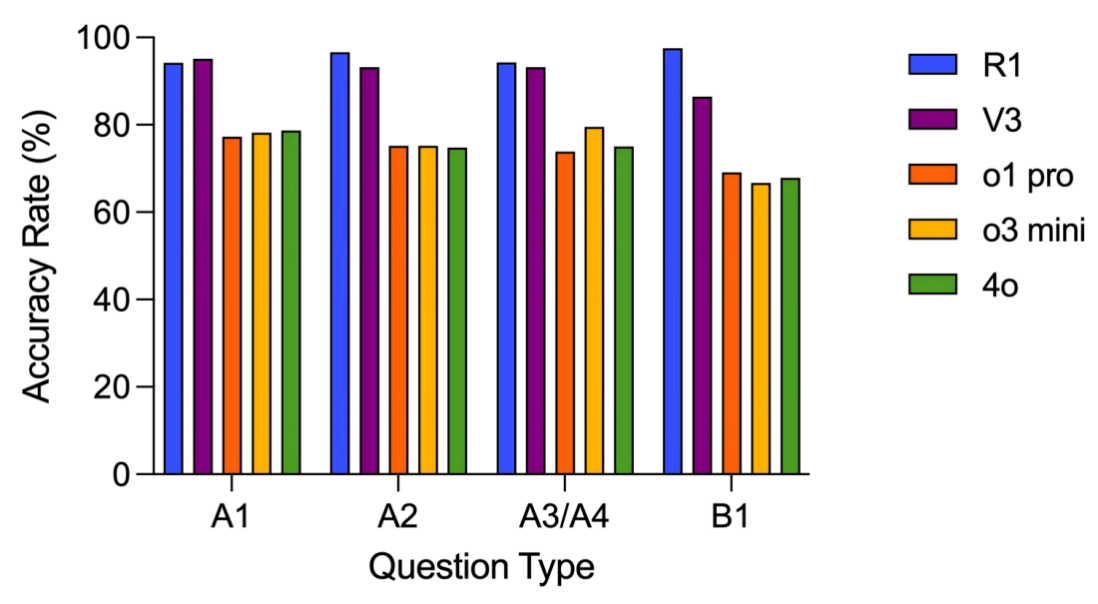
Accuracy Rates of DeepSeek-R1, DeepSeek-V3, ChatGPT-o1 pro, ChatGPT-o3 mini, and GPT-4o Across Various Question Types.

As shown in Figure 2 and Multimedia Appendix 1, for question types A1, A2, A3 and A4, and B1, DeepSeek-R1 and DeepSeek-V3 both demonstrated significantly higher accuracy than ChatGPT-o1 pro, ChatGPT-o3 mini, and GPT-4o (*P*<.05 for all comparisons). Specifically, DeepSeek-R1 achieved accuracy rates of 95.5% (212/222), 96.6% (199/206), 94% (83/88), and 98% (79/81) for the A1, A2, A3 and A4, and B1 question types, respectively. These accuracy rates were significantly higher than those of ChatGPT-o1 pro, which scored 77.3% (174/225), 75.2% (155/206), 74% (65/88), and 69% (56/81) for the same question types, respectively (*P*<.001 for all comparisons).

The performance of the 2 DeepSeek models (R1 and V3) did not show significant differences across the 4 question types (*P*>.05 for all comparisons). Similarly, the 3 OpenAI models (ChatGPT-o1 pro, ChatGPT-o3 mini, and GPT-4o) also did not demonstrate significant differences in performance across the 4 question types (*P*>.05 for all comparisons).

### Accuracy Across Case Analysis and Non–Case Analysis Questions

**Figure 3:**
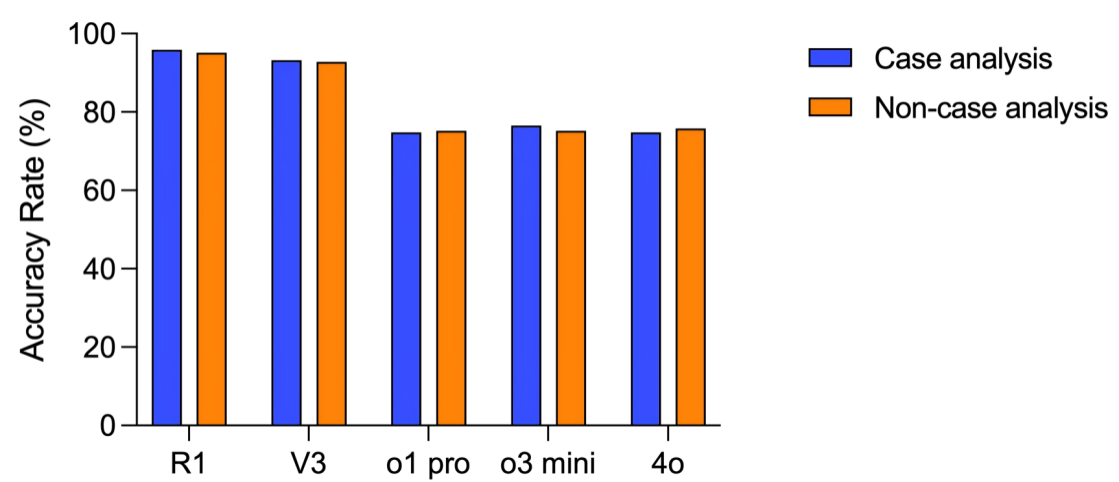
Accuracy Rates of DeepSeek-R1, DeepSeek-V3, ChatGPT-o1 pro, ChatGPT-o3 mini, and GPT-4o on Case Analysis and Non-Case Analysis Questions.

Of the 600 total questions, 294 (49%) were case analysis questions, and 306 (51%) were non–case analysis questions. As shown in Figure 3 and Multimedia Appendix 1, the accuracy rates for both case analysis and non–case analysis questions were similar across all 5 models (DeepSeek-R1, DeepSeek-V3, ChatGPT-o1 pro, ChatGPT-o3 mini, and GPT-4o).

For case analysis questions, DeepSeek-R1 and DeepSeek-V3 consistently outperformed ChatGPT-o1 pro, ChatGPT-o3 mini, and GPT-4o, with all comparisons yielding significant differences (*P*<.001 for all comparisons). Specifically, DeepSeek-R1 achieved an accuracy rate of 95.9% (282/294), which was significantly higher than the 74.8% (220/294) accuracy observed for ChatGPT-o1 pro (*P*<.001). No significant difference in accuracy was observed between DeepSeek-R1 (282/294, 95.9%) and DeepSeek-V3 (274/294, 93.2%; *P*=.10). Similarly, the 3 OpenAI models—ChatGPT-o1 pro (220/294, 74.8%), ChatGPT-o3 mini (225/294, 76.5%), and GPT-4o (220/294, 74.8%)—did not exhibit significant differences in their performance on case analysis questions (*P*>.05 for all comparisons).

For non–case analysis questions, DeepSeek-R1 and DeepSeek-V3 again demonstrated significantly higher accuracy than the OpenAI models (*P*<.001 for all comparisons). DeepSeek-R1 achieved an accuracy of 96% (291/303) for non–case analysis questions, which was significantly higher than the 75.2% (230/306) accuracy of ChatGPT-o1 pro (*P*<.001). There was no significant difference in accuracy between DeepSeek-R1 (291/303, 96%) and DeepSeek-V3 (274/294, 93.2%) on non–case analysis questions (*P*=.23). Furthermore, no significant differences were found among ChatGPT-o1 pro (230/306, 75.2%), ChatGPT-o3 mini (230/306, 75.2%), and GPT-4o (232/306, 75.8%) in terms of their performance on these questions (*P*>.05 for all comparisons).

### Performance Across Different Types of Case Analysis Questions

For the diagnosis type of case analysis question, DeepSeek-R1 achieved an accuracy of 98% (146/149), which was slightly higher than its accuracy of 92% (72/78) for treatment type of case analysis questions (*P*=.09). Similarly, ChatGPT-o3 mini showed a diagnostic accuracy of 80.5% (120/149), which was higher than its treatment accuracy of 68% (53/78; *P*=.048). DeepSeek-V3, ChatGPT-o1 pro, and GPT-4o also showed higher diagnostic accuracy compared to treatment, but the differences were not statistically significant (*P*>.05 for all comparisons).

For the examination type of case analysis question, DeepSeek-R1 achieved an accuracy rate of 98% (42/43), significantly outperforming ChatGPT-o1 pro (35/43, 81%; *P*=.02) and GPT-4o (35/43, 81%; *P*=.02). It also outperformed ChatGPT-o3 mini (37/43, 86%), although the difference was not statistically significant (*P*=.06).

Regarding diagnosis, DeepSeek-R1 and DeepSeek-V3 achieved accuracy rates of 98% (146/149) and 95.3% (142/149), respectively, both significantly outperforming the OpenAI models (accuracy rates ranging from 116/149, 77.9% to 120/149, 80.5%; *P*<.001 for all comparisons).

For treatment questions, DeepSeek-R1 and DeepSeek-V3 achieved accuracy rates of 92% (72/78) and 90% (70/78), respectively, both significantly outperforming the OpenAI models, whose accuracy ranged from 67% (52/78) to 68% (53/78; *P*<.001 for all comparisons).

**Figure 4:**
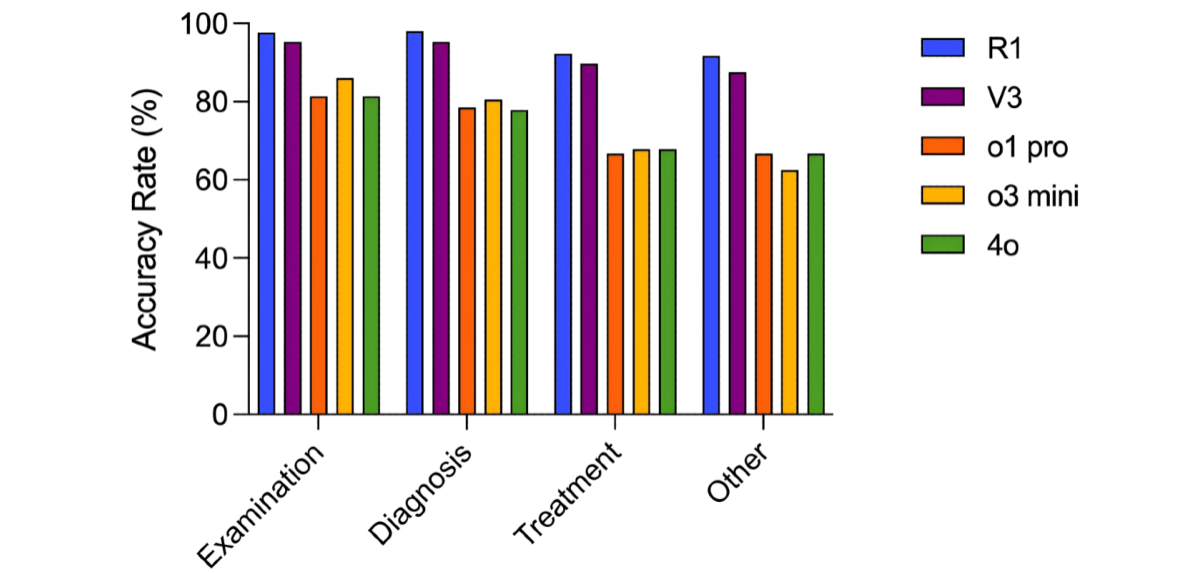
Accuracy Rates of DeepSeek-R1, DeepSeek-V3, ChatGPT-o1 pro, ChatGPT-o3 mini, and GPT-4o Across Different Types of Case Analysis Questions.

The details are shown in Figure 4 and Multimedia Appendix 1.

### Case Analysis Questions Across Different Medical Specialties

**Figure 5:**
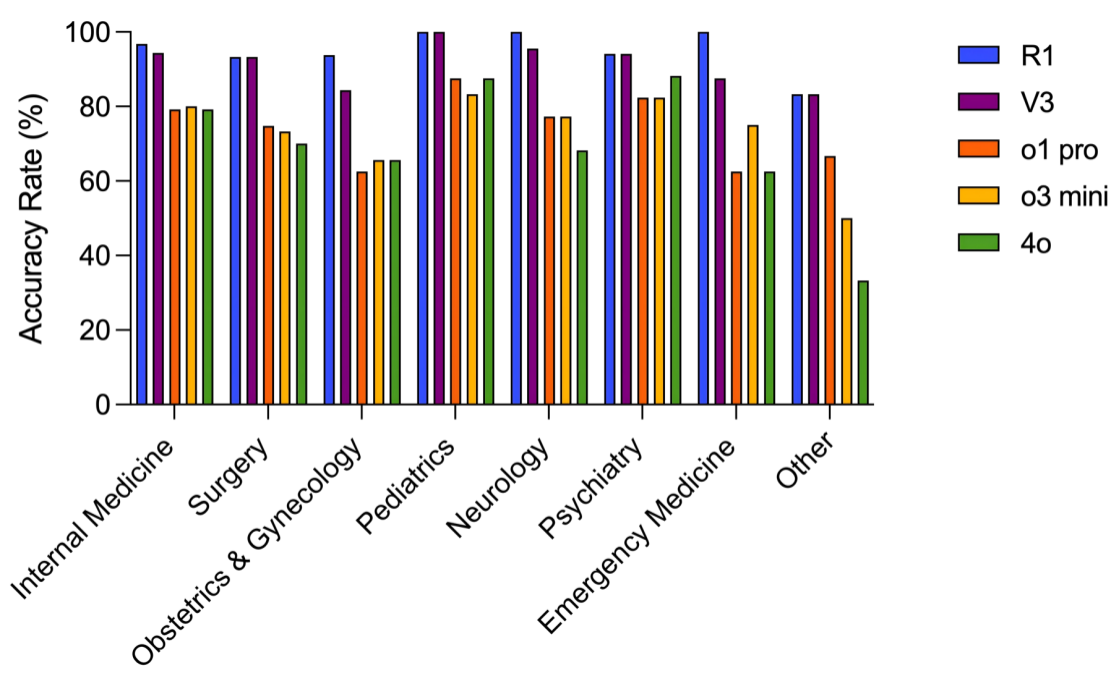
Accuracy Rates of DeepSeek-R1, DeepSeek-V3, ChatGPT-o1 pro, ChatGPT-o3 mini, and GPT-4o Across Different Medical Specialties in Case Analysis Questions.

Of the 294 case analysis questions, 125 (42.5%) were from internal medicine, 60 (20.4%) were from surgery, 32 (10.9%) were from obstetrics and gynecology, 24 (8.2%) were from pediatrics, and 22 (7.5%) were from neurology. DeepSeek-R1 achieved accuracy rates ranging from 93% (56/60) to 100% (24/24) across all specialties, whereas DeepSeek-V3 had accuracy rates between 84% (27/32) and 100% (24/24). In contrast, the OpenAI models—ChatGPT-o1 pro, ChatGPT-o3 mini, and GPT-4o—showed accuracy rates between 62% (20/32) and 88% (21/24), 66% (21/32) and 83% (20/24), and 62% (5/8) and 88% (21/24), respectively (Figure 5 and Multimedia Appendix 1).

### Accuracy Consensus

As shown in Table 1 and Multimedia Appendix 1, the accuracy consensus between DeepSeek-R1 and DeepSeek-V3 reached 97.7% (544/557), the highest among all model pairs, significantly outperforming DeepSeek-R1 alone (573/597, 96%; *P*=.04) and DeepSeek-V3 (558/600, 93%; *P*<.001). The accuracy consensus between DeepSeek-R1 and ChatGPT-o1 pro, ChatGPT-o3 mini, and GPT-4o was 95.5% (444/465), 95.9% (447/466), and 97.3% (437/449), respectively, with no significant differences compared to DeepSeek-R1’s individual accuracy (573/597, 96%; *P*>.05 for all comparisons). Regarding the 3 OpenAI models, their pairwise accuracy consensus values were 77.7% (428/551, ChatGPT-o3 mini and ChatGPT-o1 pro), 78.1% (420/538, GPT-4o and ChatGPT-o1 pro), and 79.2% (416/525, GPT-4o and ChatGPT-o3 mini), with no significant differences compared to the individual accuracy of each model (*P*>.05 for all comparisons).

The results of the κ consistency test are shown in Table 2. Specifically, the κ coefficient between ChatGPT-o1 pro and ChatGPT-o3 mini was 0.781, demonstrating excellent agreement. The κ coefficients between GPT-4o and ChatGPT-o1 pro and between GPT-4o and ChatGPT-o3 mini were 0.723 and 0.661, respectively, indicating good agreement. Notably, the κ coefficients between the 2 DeepSeek models, as well as between the DeepSeek models and the OpenAI models, were all below 0.4, suggesting poor agreement.

Our comparative analysis of accuracy consensus across different model combinations revealed that the pairing of DeepSeek-R1 and DeepSeek-V3 achieved significantly higher consensus rates than combinations consisting solely of OpenAI models (Table 1 and Multimedia Appendix 1).

**Table 1:** Accuracy consensus among DeepSeek-R1, DeepSeek-V3, ChatGPT-o1 pro, ChatGPT-o3 mini, and GPT-4o.

Model pair	Accuracy consensus, n/N (%)
DeepSeek-V3 and DeepSeek-R1	544/557 (97.7)
ChatGPT-o1 pro and DeepSeek-R1	444/465 (95.5)
ChatGPT-o1 pro and DeepSeek-V3	441/474 (93)
ChatGPT-o3 mini and DeepSeek-R1	447/466 (95.9)
ChatGPT-o3 mini and DeepSeek-V3	444/475 (93.5)
ChatGPT-o3 mini and ChatGPT-o1 pro	428/551 (77.7)
GPT-4o and DeepSeek-R1	437/449 (97.3)
GPT-4o and DeepSeek-V3	435/460 (94.6)
GPT-4o and ChatGPT-o1 pro	420/538 (78.1)
GPT-4o and ChatGPT-o3 mini	416/525 (79.2)

**Table 2:** κ coefficients for the consistency test among DeepSeek-R1, DeepSeek-V3, ChatGPT-o1 pro, ChatGPT-o3 mini, and GPT-4o.

Model pair	κ coefficient
DeepSeek-V3 and DeepSeek-R1	0.341
ChatGPT-o1 pro and DeepSeek-R1	0.171
ChatGPT-o1 pro and DeepSeek-V3	0.263
ChatGPT-o3 mini and DeepSeek-R1	0.157
ChatGPT-o3 mini and DeepSeek-V3	0.250
ChatGPT-o3 mini and ChatGPT-o1 pro	0.781
GPT-4o and DeepSeek-R1	0.066
GPT-4o and DeepSeek-V3	0.173
GPT-4o and ChatGPT-o1 pro	0.723
GPT-4o and ChatGPT-o3 mini	0.661

### English-Translated Questions

Following the translation of NMLE questions into English, GPT-4o demonstrated significantly improved performance, with an accuracy of 83.5% (501/600), representing a notable increase from its 75.3% (452/600) accuracy on the original Chinese version (*P*<.001). Nevertheless, this enhanced performance still fell significantly short of DeepSeek-R1’s 96% (573/597) accuracy on the Chinese-language questions (*P*<.001).

The performance improvement was particularly pronounced across specific question types: GPT-4o showed statistically significant gains in accuracy for A1-type (193/225, 85.8% vs 177/225, 78.7%; *P*=.03), A2-type (168/206, 81.6% vs 154/206, 74.8%; *P*=.04), and A3 and A4–type (78/88, 89% vs 66/88, 75%; *P*=.01) questions when answering English-translated versions. Notably, both case analysis and non–case analysis questions showed marked performance enhancements in the English-translated condition. The details are shown in Table 3.

**Table 3:** GPT-4o performance on Chinese-language versus English-translated questions across different question types (N=600).

Question type	Correct answers in Chinese-language questions, n (%)	Correct answers in English-translated questions, n (%)	*P* value
A1 (n=225)	177 (78.7)	193 (85.8)	.03
A2 (n=206)	154 (74.8)	168 (81.6)	.04
A3 and A4 (n=88)	66 (75)	78 (88.6)	.01
B1 (n=81)	55 (67.9)	62 (76.5)	.23
Case analysis (n=294)	220 (74.8)	246 (83.7)	<.001
Non–case analysis (n=306)	232 (75.8)	255 (83.3)	.01
Total	452 (75.3)	501 (83.5)	<.001

### Comparison of Chinese LLMs

**Figure 6:**
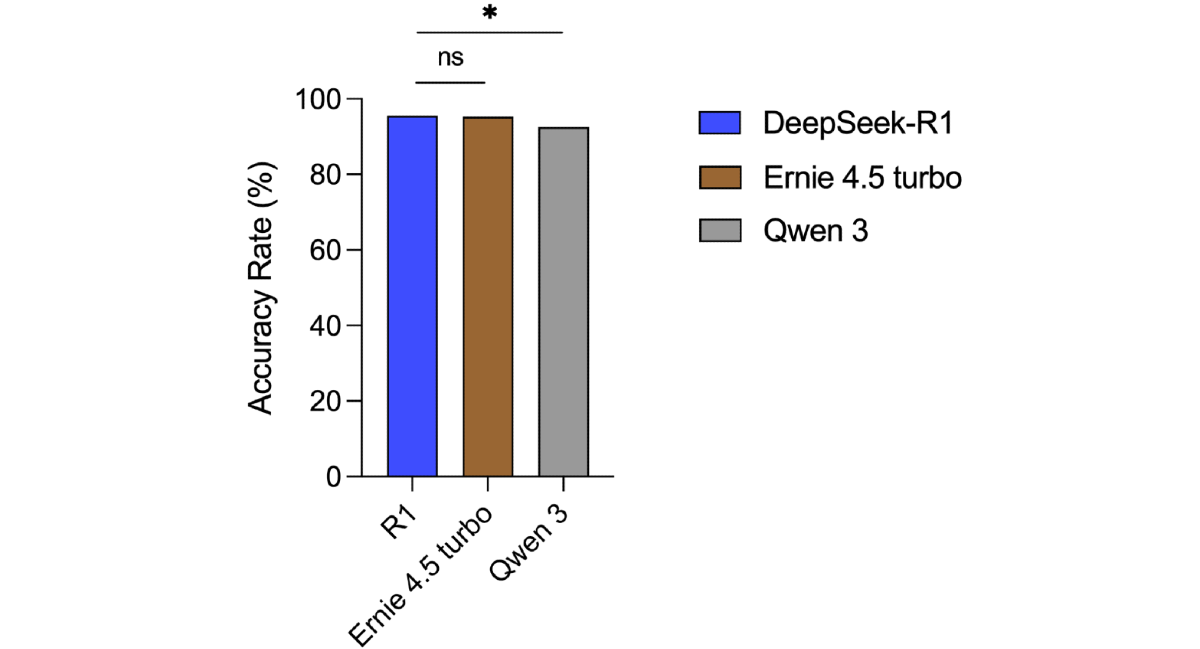
Overall accuracy rates of DeepSeek-R1, ERNIE 4.5 Turbo and Qwen 3. * *P*<.05 and ns (no significance).

We compared DeepSeek-R1 with 2 other Chinese LLMs, ERNIE 4.5 Turbo and Qwen 3, using the same NMLE questions Figure 6. ERNIE 4.5 Turbo achieved an overall accuracy of 95.3% (572/600), comparable to that of DeepSeek-R1 (573/597, 96%; *P*=.58). In contrast, Qwen 3 demonstrated an accuracy of 92.5% (555/600), significantly lower than that of DeepSeek-R1 (*P*=.01). Both ERNIE 4.5 Turbo and Qwen 3 significantly outperformed OpenAI’s 3 models (*P*<.001 for all comparisons).

## Discussion

In this study, we observed that the DeepSeek-R1 and Deepseek-V3 models achieved significantly higher accuracy rates on the NMLE, with accuracy scores of 93% (558/600) and 95.5% (212/222), respectively, substantially outperforming the OpenAI models, which demonstrated accuracy rates ranging from 75% (450/600) to 75.8% (455/600). In addition, the DeepSeek models consistently outperformed the OpenAI models across a variety of question types. A further in-depth analysis revealed that the accuracy consensus between DeepSeek-R1 and DeepSeek-V3 reached a peak of 97.7% (544/557). These findings highlight the superior performance of the DeepSeek models in the context of the NMLE when compared to OpenAI models. To the best of our knowledge, this is the first study to directly compare the performance of DeepSeek and OpenAI models within the context of a medical examination.

Previous studies have demonstrated that GPT-4’s performance on the NMLE is notably inferior to its performance on English-language exams such as the USMLE, the United Kingdom’s PLAB, and the Hong Kong Medical Licensing Examination [7,8]. Previous research has also revealed that ChatGPT was unable to pass the Taiwanese Family Medicine Board Exam in Chinese [15], indicating that OpenAI models may not perform as well on Chinese medical exams compared to their performance on their English-language counterparts. Before the advent of DeepSeek, a study found that the Chinese-developed LLM Qwen-2.5 (Alibaba Group) achieved an accuracy of 88.9% on the Chinese National Nursing Licensing Examination, surpassing GPT-4, which had an accuracy of 80.7%. Another Chinese LLM, ERNIE Bot-3.5 (Baidu), demonstrated an accuracy of 78.1%, which is comparable to that of GPT-4 [16]. Additional studies have shown that ERNIE Bot performed similarly to OpenAI’s models in recognizing Chinese health-related rumors and providing medical counseling [17,18]. Although the previous Chinese LLMs from DeepSeek had a noticeable gap compared to OpenAI models, in terms of processing Chinese medical questions, those models have already become similar to OpenAI models of the same period. In this study, another Chinese LLM—ERNIE 4.5 Turbo—performed comparably to DeepSeek in the NMLE, whereas Qwen 3, although it achieved a lower accuracy (92.5%) than DeepSeek-R1, still significantly outperformed OpenAI models.

Neither DeepSeek nor OpenAI has disclosed the exact proportion of Chinese-language data used in their models. However, as a Chinese-developed LLM, DeepSeek likely incorporates a significantly higher proportion of Chinese-language data than OpenAI’s more globally oriented models. It is estimated that OpenAI’s Chinese-language training data comprise less than 5%, whereas DeepSeek’s proportion exceeds 40%. This higher proportion of Chinese-language data likely enhances DeepSeek’s ability to understand and process Chinese-language text with greater accuracy. Our translation experiment further substantiates this hypothesis. When NMLE questions were translated into English, GPT-4o’s accuracy significantly increased from 75.3% (452/600) to 83.5% (501/600; *P*<.001). This 8.2 percentage points gain demonstrates that language barriers materially constrain OpenAI’s performance in Chinese medical examinations. Practically, these findings reveal a strategic implication: NMLE candidates using OpenAI models for preparation may improve answer reliability by translating questions into English. Nevertheless, even with optimized language conditions, GPT-4o’s translated question accuracy remained significantly inferior to DeepSeek-R1’s native-language performance (501/600, 83.5% vs 573/597, 96%; *P*<.001), suggesting that DeepSeek’s advantage extends beyond linguistic proficiency to encompass domain-specific medical knowledge alignment.

DeepSeek-R1 provides chain-of-thought (CoT) processes when answering questions, which offers us a valuable window to observe the reasoning processes of LLMs. Interestingly, DeepSeek-R1’s CoT frequently references Chinese medical textbooks, which serve as key references for the NMLE. In Multimedia Appendix 1, we present 3 specific examples of this phenomenon. In the CoT analysis of 2 case study questions, the model consistently followed a structured approach: first identifying key diagnostic elements (such as Reed-Sternberg cells in case 1 and upper gastrointestinal bleeding in case 2) and then determining the most appropriate answer choice based on medical textbooks from People’s Medical Publishing House editions. Subsequently, it systematically evaluated why alternative options were incorrect before arriving at the final correct answer. The medical textbooks published by People’s Medical Publishing House are the most important official reference materials for the NMLE. For clinically controversial questions, the examination board explicitly uses these textbooks as the definitive authority. This alignment between DeepSeek’s reference sources and the exam’s official evaluation criteria may be one of the key reasons for DeepSeek’s superior performance.

Differences in disease patterns between China and the United States, as well as variations in diagnostic criteria, treatment protocols, and clinical guidelines for the same diseases, may further contribute to DeepSeek’s superior performance on Chinese-language medical exams. For example, in this study, 8.5% (51/600) of the questions were related to tuberculosis. According to the 2024 Global Tuberculosis Report from the World Health Organization, the incidence of tuberculosis in China is approximately 13 times higher than that in the United States [19]. The higher incidence results in an abundance of Chinese-language resources related to tuberculosis, which may contribute to DeepSeek’s stronger performance on related questions.

Another important observation is that while ChatGPT-o1 pro and ChatGPT-o3 mini outperform GPT-4o in areas such as mathematics and coding [20,21], this study found that the 3 OpenAI models—ChatGPT-o1 pro, ChatGPT-o3 mini, and GPT-4o—demonstrated almost identical accuracy rates on the NMLE, with accuracy scores of 75% (450/600), 75.8% (455/600), and 75.3% (452/600), respectively. In contrast, DeepSeek-R1, which is considered superior to DeepSeek-V3 [6], achieved only a 2.5% higher accuracy than DeepSeek-V3. The primary distinction between DeepSeek-R1 and DeepSeek-V3, as well as among the OpenAI models, appears to be in their reasoning capabilities. However, the medical licensing examination is a basic-level test for physicians and does not require advanced reasoning skills. It is generally understood that case analysis questions demand more reasoning ability than non–case analysis questions. As illustrated in Figure 3, the accuracy rates for all 5 models were similar across both case analysis and non–case analysis questions. Performance differences between the 2 DeepSeek models and among the 3 OpenAI models were comparable on case analysis questions. This suggests that the reasoning abilities of both DeepSeek-V3 and GPT-4o may be adequate for a medical licensing examination. However, this also raises the possibility that the observed performance discrepancies in DeepSeek models on the NMLE are not attributable to differences in reasoning abilities but rather to the variations in the training corpora discussed previously. To further explore this, additional studies assessing DeepSeek’s performance on English-language examinations such as the USMLE would be beneficial.

DeepSeek-R1 declined to answer 3 questions, all notably related to obstetrics and gynecology (Multimedia Appendix 1). These refusals likely stem from the domain’s inherent sensitivity (pregnancy, fetal abnormalities, and reproductive rights) and associated ethical dilemmas, triggering the model’s protective filters. This conservative “silence over risk” approach aligns with DeepSeek’s technical report showing that safety reinforcement learning reduces benchmark accuracy (58.7% vs 70% without safety reinforcement learning) [22]. Such refusals represent responsible artificial intelligence (AI) design but underscore challenges in applying LLMs to high-stakes medical contexts in which comprehensive response capability remains essential.

The high accuracy of LLMs in the NMLE demonstrates their potential as auxiliary tools for exam preparation. Our findings reveal that DeepSeek models significantly outperform their OpenAI counterparts in NMLE performance, suggesting that students should prioritize DeepSeek over OpenAI models for NMLE preparation. Given that DeepSeek-R1’s accuracy improvement over DeepSeek-V3 was marginal (3%), whereas DeepSeek-V3 already achieves a high accuracy (558/600, 93%) and substantially faster response times, DeepSeek-V3 offers a balanced combination of precision and efficiency for daily practice. However, for in-depth analysis of complex clinical cases, DeepSeek-R1 may offer advantages due to its explicit CoT reasoning capabilities. When using primarily English language–trained models such as those by OpenAI, posing queries in English is recommended to mitigate performance limitations. Looking ahead, developing adaptive learning systems based on LLMs could dynamically tailor training focus according to individual error patterns observed in practice questions.

The superior performance of DeepSeek models on the NMLE suggests transformative potential for medical education. These LLMs can serve as intelligent teaching assistants, providing evidence-based tutoring with particular strength in clinical reasoning. Their case analysis capabilities enable the development of AI-standardized patients that adapt to learner levels while performance variations across question types inform personalized learning systems. As hybrid educational tools, they combine the efficiency of AI with clinical training needs, with DeepSeek-V3 offering rapid practice and DeepSeek-R1 enabling in-depth case analysis while maintaining crucial human oversight for complex decision-making.

This study has several limitations. First, it focused exclusively on the NMLE, and therefore, the reasons behind DeepSeek’s superior accuracy compared to OpenAI models cannot be conclusively determined without evaluating their performance on other English-language medical examinations such as the USMLE and PLAB. Second, the questions in this study did not include medical imaging, leaving the models’ performance on image-based medical tasks unassessed. Third, we primarily attributed DeepSeek’s superior NMLE performance to its greater amount of Chinese-language training data. However, without reliable documentation from both companies about their actual Chinese-language training data proportions, our analysis might be incomplete. The performance differences could also stem from other factors such as reasoning capabilities or DeepSeek’s tendency to reference Chinese medical textbooks.

In conclusion, all 5 models successfully passed the NMLE. DeepSeek-R1 achieved the highest accuracy rate of 96% (573/597), followed by DeepSeek-V3 at 93% (558/600). Both DeepSeek models significantly outperformed the OpenAI models, whose accuracy rates ranged from 75% (450/600) to 75.8% (455/600). DeepSeek models exhibited superior performance across various question types, including case analysis, non–case analysis, and different subtypes of case analysis questions. The accuracy consensus between DeepSeek-R1 and DeepSeek-V3 further boosted accuracy to 97.7% (544/557). The outstanding performance of DeepSeek models on the NMLE underscores their considerable potential for enhancing diagnostic and treatment decision-making, patient education, and medical popularization in Chinese-language contexts. Moreover, they represent a valuable tool for preparing for medical licensing examinations.

## Appendix

Multimedia Appendix 1 Standard prompts, model performance comparisons across question types and medical specialties, declined questions, statistical analyses, and chain-of-thought evaluation.

## Abbreviations

AI: artificial intelligence
CoT: chain-of-thought
LLM: large language model
NMLE: National Medical Licensing Examination
PLAB: Professional and Linguistic Assessments Board
USMLE: US Medical Licensing Examination
