# Supplementary material for: Evaluating the Performance of DeepSeek-R1 and DeepSeek-V3 Versus OpenAI Models in the Chinese National Medical Licensing Examination: Cross-Sectional Comparative Study
Source: JMIR Med Educ. 2025 Nov 14;11:e73469. doi: 10.2196/73469 (PMC12663704; doi:10.2196/73469)
Supplement: Multimedia Appendix 1 [file mededu_v11i1e73469_app1.doc]

**Supplementary Table 1. Standard Prompts for Different Types of Questions**

| Question Type | Prompts in Chinese | Prompts translated in English |
| --- | --- | --- |
| A1 | 答题说明：每一道考题下面有A、B、C、D、E五个备选答案，请从中选择一个最佳答案 | Answer Instructions: Each question offers five answer choices: A, B, C, D, and E. Please select the most appropriate answer. |
| A2 | 答题说明：每一道考题下面是以一个小案例出现的，其下面都有A、B、C、D、E五个备选答案，请从中选择一个最佳答案 | Answer Instructions: Each question is accompanied by a brief case scenario, followed by five answer choices: A, B, C, D, and E. Please select the most appropriate answer. |
| A3/A4 | 答题说明：以下提供若干个案例，每个案例下设若干道试题。请根据案例提供的信息，在每一道试题下面的A、B、C、D、E五个备选答案中选择一个最佳答案 | Answer Instructions: Several case scenarios are provided below, with multiple questions under each case. Based on the information presented in the case, please select the best answer from the five choices (A, B, C, D, and E) for each question. |
| B1 | 答题说明：以下提供若干组试题，每组试题共用在试题前列出的A、B、C、D、E五个备选答案，请从中选择一个 与问题关系最密切的答案。某一个备选答案可能被选择 一次、多次或不被选择。 | Answer Instructions: Several sets of questions are provided below, with each set sharing the same answer choices: A, B, C, D, and E, listed at the beginning of the questions. Please select the answer that is most relevant to each question. A particular answer choice may be selected once, multiple times, or not at all. |

**Supplementary Table 2. Performance of DeepSeek and OpenAI Models** Across Different Question Types

|  | A1 (n=225) | A2 (n=206) | A3/A4 (n=88) | B1(n=81) | Total |
| --- | --- | --- | --- | --- | --- |
| DeepSeek-R1 | 212 (95.5%)* | 199 (96.6%) | 83 (94.3%) | 79 (97.5%) | 573 (96.0%)* |
| DeepSeek-V3 | 214 (95.1%) | 192 (93.2%) | 82 (93.2%) | 70 (86.4%) | 558 (93.0%) |
| ChatGPT-o1 pro | 174 (77.3%) | 155 (75.2%) | 65 (73.9%) | 56 (69.1%) | 450 (75.0%) |
| ChatGPT-o3 mini | 176 (78.2%) | 155 (75.2%) | 70 (79.5%) | 54 (66.7%) | 455 (75.8%) |
| GPT-4o | 177 (78.7%) | 154 (74.8%) | 66 (75.0%) | 55 (67.9%) | 452 (75.3%) |
| P | < .001 | < .001 | < .001 | < .001 | < .001 |

***** Three questions unanswered by DeepSeek-R1 were treated as omissions and excluded from analysis

Supplementary Table 3. Three Questions Declined by DeepSeek-R1 and Comparative Model Responses

| Question & Options (English Translation) | Correct Answer | Response of Different Models | | | | |
| --- | --- | --- | --- | --- | --- | --- |
|  |  | DeepSeek-R1 | DeepSeek-V3 | ChatGPT-o1 | ChatGPT-o3 | GPT-4o |
| The correct description of clinical manifestations of early miscarriage is:  A. The size of the uterus in missed abortion corresponds to the weeks of amenorrhea.  B. In complete abortion, the cervix is closed, and the uterus is close to normal size.  C. In early miscarriage, abdominal pain occurs before vaginal bleeding.  D. In inevitable abortion, the cervical os has not yet dilated.  E. In incomplete abortion, the size of the uterus corresponds to the weeks of amenorrhea. | B | Sorry, I haven’t yet learned how to approach these types of questions. I specialize in math, coding, and logic-related problems. Feel free to engage with me on these topics | B | B | B | B |
| The most common symptom of a molar pregnancy is:  A. Abnormal uterine enlargement  B. Pregnancy-related vomiting  C. Vaginal bleeding after missed menstruation  D. Irregular lower abdominal pain  E. Grape-like tissue passing through the vagina | C | Sorry, I haven’t yet learned how to approach these types of questions. I specialize in math, coding, and logic-related problems. Feel free to engage with me on these topics | C | C | C | C |
| The commonly used medications for endocrine therapy in endometrial cancer are:  A. Estrogen  B. Progesterone  C. Androgen  D. Glucocorticoids  E. GnRH-a | B | Sorry, I haven’t yet learned how to approach these types of questions. I specialize in math, coding, and logic-related problems. Feel free to engage with me on these topics | B | B | B | B |

**Supplementary Table 4. Performance of DeepSeek and OpenAI Models on Case Analysis and Non-Case Analysis Questions.**

|  | Case analysis（n=294） | Non-Case Analysis（n=306） | P |
| --- | --- | --- | --- |
| DeepSeek-R1 | 282 (95.9%) | 291 (96.0%)* | .628 |
| DeepSeek-V3 | 274 (93.2%) | 284 (92.8%) | .853 |
| ChatGPT-o1 pro | 220 (74.8%) | 230 (75.2%) | .925 |
| ChatGPT-o3 mini | 225 (76.5%) | 230 (75.2%) | .696 |
| GPT-4o | 220 (74.8%) | 232 (75.8%) | .779 |
| P | < .001 | < .001 |  |

***** Three questions unanswered by DeepSeek-R1 were treated as omissions and excluded from analysis

**Supplementary Table 5. Performance of DeepSeek and OpenAI Models Across Different Types of Case Analysis Questions.**

|  | Examination (n=43) | Diagnosis (n=149) | Treatment (n=78) | Other (n=24) | P |
| --- | --- | --- | --- | --- | --- |
| DeepSeek-R1 | 42 (98%) | 146 (98%) | 72 (92%) | 22(92%) | .149 |
| DeepSeek-V3 | 41 (95%) | 142 (95%) | 70 (90%) | 21(88%) | .285 |
| ChatGPT-o1 pro | 35 (81%) | 117 (79%) | 52 (67%) | 16 (67%) | .099 |
| ChatGPT-o3 mini | 37 (86%) | 120 (81%) | 53 (68%) | 15 (63%) | .037 |
| GPT-4o | 35 (81%) | 116 (78%) | 53 (68%) | 16 (67%) | .211 |
| P | .003 | <.001 | <.001 | .053 |  |

**Supplementary Table 6. Performance of DeepSeek and OpenAI Models Across Different Medical Specialties in Case Analysis Questions.**

|  | Internal Medicine (n=125) | Surgery (n=60) | Obstetrics & Gynecology (n=32) | Pediatrics (n=24) | Neurology (n=22) | Psychiatry (n=17) | Emergency Medicine (n=8) | Other (n=6) |
| --- | --- | --- | --- | --- | --- | --- | --- | --- |
| DeepSeek-R1 | 121  (97%) | 56 (93%) | 30  (94%) | 24 (100%) | 22 (100%) | 16 (94%) | 8 (100%) | 5 (83%) |
| DeepSeek-V3 | 118 (94%) | 56 (93%) | 27  (84%) | 24 (100%) | 21 (96%) | 16 (94%) | 7  (88%) | 5 (83%) |
| ChatGPT-o1 pro | 99 (79%) | 40 (74%) | 20  (63%) | 21 (88%) | 17 (77%) | 14 (82%) | 5  (63%) | 4 (67%) |
| ChatGPT-o3 mini | 100 (80%) | 44 (73%) | 21  (66%) | 20 (83%) | 17 (77%) | 14 (82%) | 6  (75%) | 3 (50%) |
| GPT-4o | 99 (79%) | 42 (70%) | 21 (66%) | 21  (88%) | 15 (68%) | 15 (88%) | 5  (63%) | 2 (33%) |

**Supplementary Table 7. Pairwise Statistical Comparisons of Accuracy Consensus Between Model Combinations (P-values)**

|  | R1+ V3 | R1+o1 | R1+o3 | R1+4o | V3+o1 | V3+o3 | V3+4o | o1+o3 | o1+4o |
| --- | --- | --- | --- | --- | --- | --- | --- | --- | --- |
| R1+o1 | .053 |  |  |  |  |  |  |  |  |
| R1+o3 | .111 | .741 |  |  |  |  |  |  |  |
| R1+4o | .732 | .135 | .240 |  |  |  |  |  |  |
| V3+o1 | <.001 | .107 | .053 | .002 |  |  |  |  |  |
| V3+o3 | .001 | .178 | .094 | .005 | .789 |  |  |  |  |
| V3+4o | .009 | .520 | .332 | .035 | .334 | .482 |  |  |  |
| o1+o3 | < .001 | < .001 | < .001 | < .001 | < .001 | < .001 | < .001 |  |  |
| o1+4o | < .001 | < .001 | < .001 | < .001 | < .001 | < .001 | < .001 | .877 |  |
| o3+4o | < .001 | < .001 | < .001 | < .001 | < .001 | < .001 | < .001 | .534 | .641 |

Abbreviations: R1:Deepseek-R1;V3: Deepseek-V3; o1: ChatGPT-o1 pro; o3: ChatGPT-o3 mini; 4o: GPT-4o.

**Supplementary Results: Case of Chain of Thought**

**Case 1**

**Question in Translated in English**

A 10-year-old girl presented with cervical lymphadenopathy for 2 weeks. Biopsy revealed destruction of lymph node architecture and the presence of Reed-Sternberg (R-S) cells. Diagnosis Options: A. Infectious mononucleosis; B. Reactive lymph node hyperplasia; C. Hodgkin lymphoma; D. Non-Hodgkin lymphoma; E. Tuberculous lymphadenitis; Chain-of-Thought in Chinese

**DeepSeek-R1：Chain-of-Thought Translated in English**

The question is: Female, 10 years old. Swollen neck lymph nodes for 2 weeks; biopsy shows destruction of lymph node architecture and presence of Reed-Sternberg (R-S) cells. What is the diagnosis? The options are A. Infectious mononucleosis, B. Reactive lymphadenopathy, C. Hodgkin lymphoma, D. Non-Hodgkin lymphoma, E. Tuberculosis of the lymph nodes.

Key point: The biopsy findings show destruction of the lymph node structure and the presence of R-S cells. R-S cells are characteristic of Hodgkin lymphoma, **according to the 9th edition of Pathology and the 9th edition of Internal Medicine**.

Recalling the diagnostic criteria for Hodgkin lymphoma: One of the major pathological features of Hodgkin lymphoma is the presence of Reed-Sternberg cells, which are typically found in lymph node biopsies and are accompanied by destruction of the lymph node architecture.

Now, reviewing the other options:

A. Infectious mononucleosis: This is usually caused by the Epstein-Barr virus and presents with symptoms such as fever, pharyngitis, and swollen lymph nodes. However, R-S cells do not appear in a biopsy; it may show non-specific reactive hyperplasia.

B. Reactive lymphadenopathy: This is a benign condition where the lymph node structure is usually preserved, and there are no R-S cells.

C. Hodgkin lymphoma: This directly matches, as R-S cells are the hallmark of Hodgkin lymphoma.

D. Non-Hodgkin lymphoma: There are many types of non-Hodgkin lymphoma, but they do not typically contain R-S cells; these cells are specific to Hodgkin lymphoma. Non-Hodgkin lymphoma may have other types of abnormal cells.

E. Tuberculosis of the lymph nodes: Tuberculosis can cause swollen lymph nodes and structural damage, but R-S cells are not present; it may exhibit caseous necrosis or acid-fast bacilli.

The patient is a 10-year-old girl with swollen neck lymph nodes for 2 weeks. Hodgkin lymphoma can occur in children, especially adolescents and young adults. Swelling of the neck lymph nodes is a common presentation.

In infectious mononucleosis, swollen lymph nodes are common, but a biopsy usually reveals reactive changes rather than structural destruction or R-S cells. R-S cells are specific to Hodgkin lymphoma.

Therefore, based on the biopsy findings of lymph node architectural destruction and the presence of R-S cells, the best diagnosis is Hodgkin lymphoma.

Confirming R-S cells: Reed-Sternberg cells are large binucleated or multinucleated cells with prominent nucleoli and are the pathological hallmark of Hodgkin lymphoma.

In non-Hodgkin lymphoma, the cytological features are different and do not include typical R-S cells.

Thus, the answer should be C. Hodgkin lymphoma.

**Case 2**

**Question in Translated in English**

A 25-year-old male presented with epigastric pain on an empty stomach and acid reflux for over one month. Two hours prior, he vomited approximately 200 mL of coffee-ground-like material. There was no significant weight change recently, and he denied any history of chronic liver disease. Physical examination: signs of anemia; soft abdomen; tenderness in the upper abdomen; no rebound tenderness; liver and spleen not palpable under the costal margin. Most Appropriate Medication: A. Tranexamic acid; B. Famotidine; C. Omeprazole; D. Thrombin; E. Pituitrin.

**Chain-of-Thought Translated in English**

First, the question is: Male, 25 years old. Epigastric pain when hungry accompanied by acid reflux for more than one month. Vomited coffee-ground fluid once two hours ago, amount about 200 ml. No significant change in weight recently, denies history of chronic liver disease. Physical examination: anemic appearance, soft abdomen, upper abdominal tenderness, no rebound tenderness, liver and spleen not palpable below the costal margin. The most appropriate drug is? The options are A. Tranexamic acid, B. Famotidine, C. Omeprazole, D. Thrombin, E. Posterior pituitary extract.

Key symptoms and signs:

Epigastric pain when hungry accompanied by acid reflux for more than one month: this suggests possible peptic ulcer disease, especially duodenal ulcer, because hunger pain is a typical presentation.

Vomiting coffee-ground fluid: this indicates upper gastrointestinal bleeding; the coffee-ground appearance is usually due to blood being altered by gastric acid, indicating the bleeding source is from the upper digestive tract.

Volume about 200 ml: moderate amount of bleeding.

No significant recent weight loss: no obvious cachexia, possibly not malignant tumor.

Denies history of chronic liver disease: rules out bleeding caused by esophageal varices due to cirrhosis.

Physical findings: anemic appearance (indicates blood loss), upper abdominal tenderness, no rebound tenderness (no peritonitis), liver and spleen not palpable (no hepatosplenomegaly).

Taken together, the most likely diagnosis is upper gastrointestinal bleeding caused by peptic ulcer, especially duodenal ulcer, because hunger pain and acid reflux are common symptoms.

Now, the question asks "the most appropriate drug," meaning which medication should be the first-line treatment for the current condition.

**According to Chinese medical textbooks such as the 9th edition of *Internal Medicine*, the management principles for upper gastrointestinal bleeding are:**

For bleeding caused by peptic ulcer, the first choice is proton pump inhibitors (such as omeprazole), because they can effectively inhibit gastric acid, promote ulcer healing and hemostasis.

Endoscopic therapy is the gold standard, but drug treatment is the basis.

H2 receptor antagonists (such as famotidine) are less effective than PPIs and have now become second-line agents.

Hemostatic drugs such as tranexamic acid and thrombin have limited roles in ulcer bleeding, with insufficient evidence.

Posterior pituitary extract is mainly used for esophagogastric variceal bleeding.

In this case, the patient has epigastric pain when hungry and acid reflux, suggesting ulcer; vomiting coffee-ground fluid indicates upper gastrointestinal bleeding; no history of liver disease excludes varices. Therefore, the most likely cause is peptic ulcer bleeding.

The most suitable drug should be omeprazole, because it can rapidly increase intragastric pH, reduce acid erosion at the bleeding site, and promote hemostasis.

Other options:

Tranexamic acid: may be used as adjuvant, but not the first choice.

Famotidine: H2 receptor antagonist, acid suppression effect is inferior to PPIs.

Thrombin: local action, oral administration may be destroyed by gastric acid, poor efficacy.

Posterior pituitary extract: for varices, not applicable.

Therefore, the best answer should be C. Omeprazole.

**Case 3**

**Question in Translated in English**

Gastrointestinal malignancies commonly metastasize via hematogenous spread to which of the following sites? A. Liver; B. Lung; C. Bone; D. Brain; E. Kidney.

**Chain-of-Thought Translated in English**

First, the question is: Gastrointestinal malignancies commonly metastasize via hematogenous spread to which site? The options are A. Liver, B. Lung, C. Bone, D. Brain, E. Kidney.

I need to recall the metastatic pathways of gastrointestinal malignancies. Gastrointestinal malignancies include gastric cancer, colorectal cancer, etc., and they can metastasize through the bloodstream (hematogenous metastasis) to other organs.

Hematogenous metastasis means that cancer cells spread through the blood to other parts of the body. The common metastatic sites depend on the location of the primary tumor and the direction of blood flow.

For gastrointestinal malignancies, especially colorectal cancer, the liver is the most common site of hematogenous metastasis. This is because the venous drainage of the gastrointestinal tract mainly enters the liver through the portal venous system. The portal vein collects blood from the intestines and directly delivers it to the liver, so cancer cells easily metastasize to the liver first.

For example, in colorectal cancer, the liver is the most common site of hematogenous metastasis. Statistical data show that up to 50% of colorectal cancer patients develop liver metastases at diagnosis or during the course of the disease.

Other options:

B. Lung: The lung is also a common metastatic site because blood from the whole body returns to the heart through the vena cava and then flows to the lungs. However, for gastrointestinal tumors, especially lower digestive tract tumors, the cancer cells may first reach the liver, and then from the liver through the hepatic veins to the lungs. Therefore, lung metastasis is also common, but may not be as common as liver metastasis.

C. Bone: Bone metastasis is rare in gastrointestinal tumors and more common in prostate cancer, breast cancer, etc.

D. Brain: Brain metastasis is not the most common in gastrointestinal tumors and is more frequently seen in lung cancer, breast cancer, etc.

E. Kidney: Renal metastasis is uncommon in malignant tumors, and the kidney is not a common metastatic site.

According to medical knowledge:

The most common site of hematogenous metastasis in gastrointestinal malignancies is the liver, due to the portal venous system.

The second most common is the lung, as cancer cells can enter the systemic circulation from the liver.

Metastasis to bone, brain, and kidney is relatively rare in gastrointestinal tumors.

**In the 9th edition of *Pathology* and the 9th edition of *Surgery*, when discussing tumor metastasis, the frequency of liver metastasis in gastrointestinal tumors is emphasized.**

**For example, in *Pathology* (People's Health Publishing House edition), when describing tumor metastasis pathways, it points out that intra-abdominal tumors often metastasize to the liver through the portal vein.**

Therefore, for this question, the best answer should be A. Liver.

Confirming other options:

B. Lung: Lung metastasis is also common in colorectal cancer, but liver metastasis is more common, especially initial metastasis.

C. Bone: Bone metastasis occurs less frequently in gastrointestinal tumors.

D. Brain: Similarly, not the most common.

E. Kidney: Renal metastasis is rare.

Therefore, the most common metastatic site is the liver.
